# The Influence of Parents on Medication Adherence of Their Children in China: A Cross-Sectional Online Investigation Based on Health Belief Model

**DOI:** 10.3389/fpubh.2022.845032

**Published:** 2022-04-14

**Authors:** Pu Ge, Si-tong Liu, Shu-xian Xu, Jin-zi Zhang, Yong-jie Lai, Run-chen Fu, Xin-yu Ke, Juan Zhao, Ying Bian, Yi-bo Wu

**Affiliations:** ^1^Institute of Chinese Medical Sciences, University of Macau, Macau, China; ^2^State Key Laboratory of Quality Research in Chinese Medicine, University of Macau, Macau, China; ^3^Department of Public Health and Medicinal Administration, Faculty of Health Sciences, University of Macau, Macau, China; ^4^School of Pharmacy, Peking University, Beijing, China; ^5^School of Pharmacy, Shenyang Pharmaceutical University, Shenyang, China; ^6^College of Humanities and Social Sciences, Harbin Medical University, Harbin, China; ^7^Cheeloo College of Medicine, Shandong University, Jinan, China; ^8^School of Pharmacy, Shandong First Medical University, Taian, China; ^9^The Fourth Hospital of Harbin, Harbin, China; ^10^School of Public Health, Peking University, Beijing, China

**Keywords:** medication adherence, children, health belief model, influencing factors, structural

## Abstract

**Objective:**

To explore the influence of parents on the medication adherence of their children.

**Study Design:**

A cross-sectional online investigation.

**Methods:**

A questionnaire with 41 questions was designed based on the health belief model (HBM) distributed and collected online in 28 cities around China through multi-stage stratified sampling. The reliability of the questionnaire was assessed with Cronbach's α coefficient and split-half reliability, and its validity was evaluated with exploratory factor analysis and content validity index. The structural equation model (SEM) was constructed to explore the relationship between the parents' health beliefs and their children's medication adherence. Subgroup analysis was conducted to study the differences between parents with different demographic characteristics (male and female, rural and urban).

**Results:**

573 questionnaires were included for analysis, with an effective rate of 62.97%. The Cronbach'α coefficient of the questionnaire was 0.821 > 0.6, the split-half reliability was 0.651 > 0.6, the I-CVI of each dimension were >0.78, and the S-CVI/AVE (I-CVI average) was 0.95 > 0.9. The result of the questionnaire exploratory factor analysis met the standard. According to the SEM, self-efficacy (λ = 0.177), perceived susceptibility (λ = −0.244), and perceived severity (λ = 0.243) were direct influencing factors of children's medication adherence. In the subgroup analysis, the model established by each subgroup was consistent with the model established by the overall sample. The absolute values of females' perceived susceptibility, severity, and self-efficacy for their children's medication adherence path coefficients were higher than males'.

**Conclusion:**

Parents' perceived severity and self-efficacy may positively impact on their children's medication adherence, while parents' susceptibility to children's medication non-adherence may negatively impact on children's medication adherence. Objective constraints, perceived barriers, and benefits may in directly impact on children's medication adherence. Women's health beliefs appear to have a more significant impact on their children's medication adherence than men's. It may be an effective strategy to increase their children's medication adherence by improving parents' health beliefs. Medical staff should explain medication adherence knowledge to the parents of children, and inform the children of the possible consequences of non-adherence with medication, to improve the subjective perception of parents on the severity of children's non-adherence with medication, and improve parents' self-efficacy in rational medication for children. In addition, attention should be paid to the mental health of the parents, and more social and psychological support.

## Introduction

In the field of medicine, patients' adherence is used to describe how a patient correctly follows medical advice. It most commonly refers to medication or drug adherence, but it can also apply to other situations such as medical device use, self-care, and self-directed exercises (1). The medication adherence of children refers to the consistency between the medication behaviors of children and the doctors' prescriptions, and is influenced by patients, healthcare providers, and caregivers (2). In other words, it means the degree of implementation of the drug treatment plan by children, which significantly affects therapeutic efficacy and disease prognosis. Children taking medicines following the doctor's orders can effectively control the condition, avoid related complications, and prevent adverse events. Poor medication adherence of children may lead to severe problems such as impaired physical and mental health, increased family burden, waste of medical resources, and so on (3–5). Medication adherence can be problematic for the pediatric population. Rates of children's medication adherence are highly variable, ranging from 11 to 93%, with an estimated average of around 50% (6). Non-adherence in pediatric patients increases the number of emergency department visits and hospitalizations and is costly to the healthcare system. Seven studies showed that non-adherent children and adolescents had increased emergency department visits (3). Therefore, it is essential to explore the related influencing factors of children's medication adherence. Further investigation are required to improve adherence in children reducing overall healthcare costs and possibly decreasing morbidity and mortality resulting from non-adherence (3). Many studies have shown that children's medication adherence is influenced by factors such as disease type, drugs, parent's intervention, children's psychology (7), and so on. Among them, parents have a direct and significant influence on children's medication adherence (8).

Children are a unique and heterogeneous group of patientswhose body is responses to drugs are different from adults', and their medication adherence may depend entirely on others (9). Children's supervisor refers to the person in charge of children's medication in the family, including parents, grandparents, uncles, aunts, brothers, sisters, etc. Numerous studies have identified factors influencing children's medication adherence, including the general sociological characteristics of supervisors' education level (10), family income (11), number of children (12), occupation (12), health belief (9), knowledge of diseases and drugs (9), belief and worry about the necessity of drugs (13), etc., For example, in families with non-only children, the family members may be unable to supervise the children's medication in time, which leads to poor adherence and difficulty in medication. Occupations of family members as manual laborers or free/full-time employment may easily lead to difficulties in children's medication use.

Furthermore, this study it is considered that family members' perspectives on diseases may affect children's perceptions of diseases, and their inadequate awareness, ignorance of the importance of medication, or failure to timely supervise children's medication may all affect children's medication adherence, leading to medication difficulties (12). Caregivers think that their children take too many drugs, which will increase the risk of children suffering from long-term effects, such as drug addiction or losing efficacy. In a study for children with asthma, some caregivers think that the combination medication is too much for the children, so they only give the children controller medication when they have asthma symptoms, even though the prescription was suggested to use medication every day (9). Recently, a bibliometric study found that children's adherence to antibiotics, anti-asthma, and anti-epileptic drugs is being extensively studied (14).

## Theoretical Basis and Research Hypotheses

The health belief model (HBM) is a critical theory that considers the influence of personal factors on behavior from the perspective of people's health beliefs. It emphasizes individuals' attitudes and beliefs about a specific problem, and the benefits and obstacles of taking preventive actions may lead to specific behaviors (15). The health belief model is widely used to predict health behaviors and explain the changes in health-related behaviors (16). The health belief model usually includes five dimensions: perceived susceptibility, perceived severity, perceived benefits, perceived obstacles, and self-efficacy (17). Among them, perceived susceptibility describes people's subjective belief that they know the risk of negative health outcomes in some cases. This studyrefers to the possibility that parents perceive their children's poor medication adherence. In a study on the susceptibility of high-risk patients to chronic kidney disease in primary health care practice, compared with participants with higher health literacy, participants with lower health literacy have lower susceptibility to chronic kidney disease (18). Perceived severity refers to an individual's subjective perception of potential hazards. This study refers to the severity of adverse consequences caused by parents' belief that their children do not comply with medication. Patients with coronary heart disease are more inclined to follow the doctor's advice, take medicine with higher adherence, and pay more attention to a healthy lifestyle when they are aware of the severe harm of the disease to health or think that they are more likely to have myocardial infarction (19). Perceived benefits refer to people's prediction of the benefits of taking healthy behaviors. The more people are aware of the benefits of taking healthy behaviors, the more likely they will do so. Here, it refers to parents' perception of the health benefits brought by their children's medication adherence. A study on the related factors of clinical nurses' hand-washing practice based on health belief model found that perceived benefits were positively correlated with clinical nurses' hand-washing habits(20). Perception barrier refers to the subjective prediction of obstacles that individuals may encounter in taking healthy behaviors (21). The more obstacles people feel in taking healthy behaviors, the harder it is to get them to adopt healthy behaviors. This study refers to the obstacles that parents think their children may have in adhering to medical treatment. Self-efficacy refers to a person's subjective judgment on whether he can successfully carry out a certain behavior. The higher the self-efficacy, the more likely he will take a behavior beneficial to his health and stick to it. This study refers to whether parents think they can take measures to make their children comply with medication. In a study on the adherence behavior of patients with coronary heart disease in the community, it was found that individuals with higher self-efficacy and less cognitive impairment were more likely to follow the doctor's advice (19). In this study, we defined other objective factors that may affect children's medication adherence as objective constraints, which were also included in our study. For example, a study found two key health system obstacles for adequate care and management of type 2 diabetes: the financial constraints faced by patients and the limited access to medical services and drugs (22).

Previous studies on children's adherence mostly explored the influencing factors of children's medication adherence from many angles, such as simultaneously exploring various factors such as disease types, drug dosage forms, children's age, psychology, parents, etc., (5, 12). Few studies specifically explored the influence of parents' factors on children's medication adherence. The subjects of HBM-based compliance studies are mostly adult patients, such as those with hypertension and diabetes (23, 24). Most literature on the influencing factors of children's medication adherence can only analyze the correlation between influencing factors and children's medication adherence but fail to explore the causal relationship between the two.

Based on the limitations of previous studies, this study aims to determine the influence of parents' health beliefs on children's medication adherence by using a structural equation model (SEM) based on HBM and provide direction and motivation for future research on improving children's medication adherence and making targeted suggestions.

**Figure 1:**
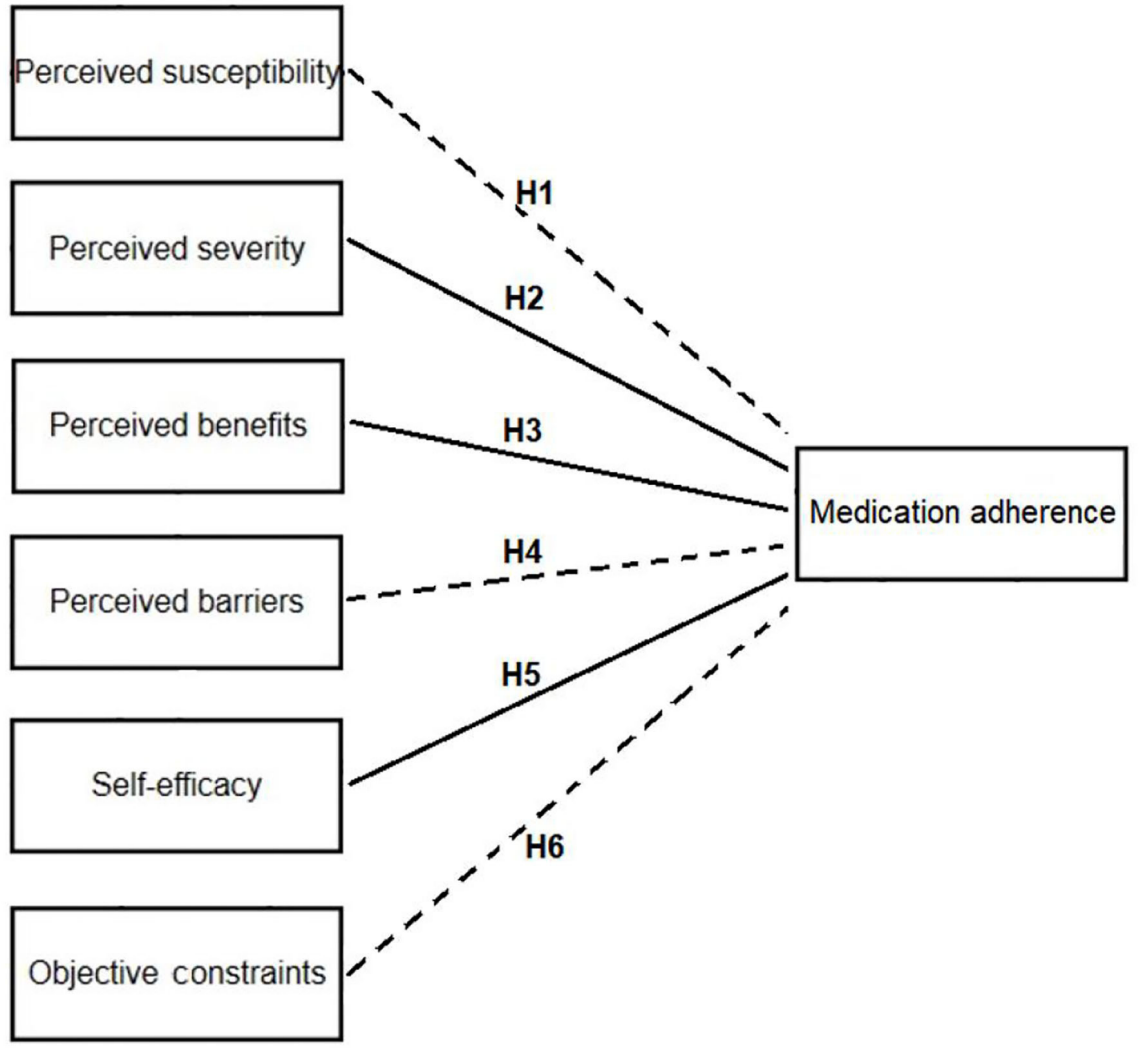
Research model diagram of the relationship between parents' health beliefs and their children's medication adherence. H1–H6 are six hypotheses of this study: H1: parents' perceived susceptibility has a negative impact on their children's medication adherence. H2: parents' perceived severity has a positive impact on their children's medication adherence. H3: parents' perceived benefits have a positive impact on their children's medication adherence. H4: parents' perception disorder has a negative impact on their children's medication adherence. H5: parents' self-efficacy has a positive impact on their children's medication adherence. H6: parents' objective constraints have a negative impact on their children's medication adherence.

Based on the above analysis, we put forward the following assumptions in this study (Figure 1):

H1: Parents' perceived susceptibility has a negative impact on their children's medication adherence.

H2: Parents' perceived severity has a positive impact on their children's medication adherence.

H3: Parents' perceived benefits have a positive impact on their children's medication adherence.

H4: Parents' perception disorder has a negative impact on their children's medication adherence.

H5: Parents' self-efficacy has a positive impact on their children's medication adherence.

H6: Parents' objective constraints have a negative impact on their children's medication adherence.

## Materials and Methods

### Participants

Inclusion criteria: (1) Parents aged 21 years and above and those who are responsible for the medication of children in their family; (2) The age of the respondents' children should be below 12 years old; (3) Parents who agree to participate in this research.

Exclusion criteria: (1) Medical faculty (doctors, pharmacists, nurses, etc.). (2) Parents who are suffering from severe mental, hearing, and visual impairment and those who cannot think independently or express clearly. (3) Parents whose children have chronic diseases (hypertension, diabetes, congenital heart disease, asthma, chronic kidney disease, epilepsy, etc.,). Due to the relatively more types and times of medication for children with chronic disease, the complex factors affecting their medication adherence and the relative difficulty in online recruitment of parents of children with chronic disease, this study only studied the medication adherence of children with the non-chronic disease. Before the parents filled out the questionnaire, the investigator would first ask whether their children suffered from chronic diseases, including hypertension, epilepsy, diabetes, asthma, chronic kidney disease, congenital heart disease, etc. If a subject answered “yes”, the investigator would not conduct further investigation, whichmeans the subject would be excluded from the study.

### Design of the Questionnaire

The questionnaire was designed based on HBM. HBM hypothesizes is that whether a person adopts health behaviors is mainly related to six factors: perceived susceptibility, perceived severity, perceived benefits of the health behavior, perceived barriers of the health behavior, cues to action, and self-efficacy. The questionnaire included four parts with 41 questions. The first part was informed consent. The second part was the basic information of the respondent, including gender, age, province, education background, marital status, number of children under care, domicile (urban or rural), average monthly income per person in the family, which included 8 questions. The third part was a self-administered questionnaire based on the health beliefs model, which included 26 items (17, 25–27) (see Table 1 for details). Each item adopted a Likert 5-level scoring method ranging from l to 5 points. This part was divided into six dimensions, which were perceived severity (5 items) (e.g., If my children take medicine without following the doctor's advice, the effectiveness of the medication may be reduced), perceived benefits (3 items) (e.g., I believe that taking drugs under medical supervision contribute to children's control and improvement of diseases), self-efficacy (3 items) (e.g., I am able to administer drugs to my children under medical supervision), perceived barriers (7 items) (e.g., The drug instructions are too complicated to understand the rational application, dosage and notes of medication), objective constraints (4 items) (e.g., Medical resources and healthcare environment affect children's medication adherence), and perceived susceptibility (4 items) (e.g., My children are more likely to take medicine without following doctor's prescribed dosage than other children). The fourth part was a tool for evaluating the medication adherence of children taken care of by the respondents (see Table 1 for details), which consisted of 7 items. Each item was scored 1–2 points, with a maximum of 14 points. This part included the supervisors' reaction when the children's condition improved or worsened, whether the supervisor gave the children the medication at the time and dosage prescribed by the doctor and their reaction when their children forgot to take the drug. We have made the following regulations by referring to the scoring methods of existing related studies, expert consultation, and pre-research. Subjects with a 13–14 on the medication adherence assessment tool had good medication adherence in their children. Subjects with a score of 12 had moderate medication adherence in their children. Subjects with a score of 11 and below had poor medication adherence in their children. All the items in the questionnaire were designed according to the relevant literature and personal practical experience in combination with expert consultation.

**Table 1:** Questionnaire structure for health belief section.

**Dimension**	**No. of items**	**Example**
Perceived susceptibility	4	My children are more likely to take medicine without following the doctor's prescribed dosage than other children.
Perceived severity	5	If my children take medicine without following the doctor's advice, the effectiveness of the medication may be reduced.
Perceived benefits	3	I believe that taking drugs under medical supervision contribute to children's control and improvement of diseases.
Perceived barriers	7	The drug instructions are too complicated to understand the rational application, dosage, and medication notes.
Self-efficacy	3	I can administer drugs to my children under medical supervision.
Objective constraints	4	Medical resources and the healthcare environment affect children's medication adherence.
Medication adherence of children	7	When my child is sick, if its condition gets worse or less, I will take it to the doctor again and follow the doctor's orders to change the type and dose of medication.

### Sampling Method

**Figure 2:**
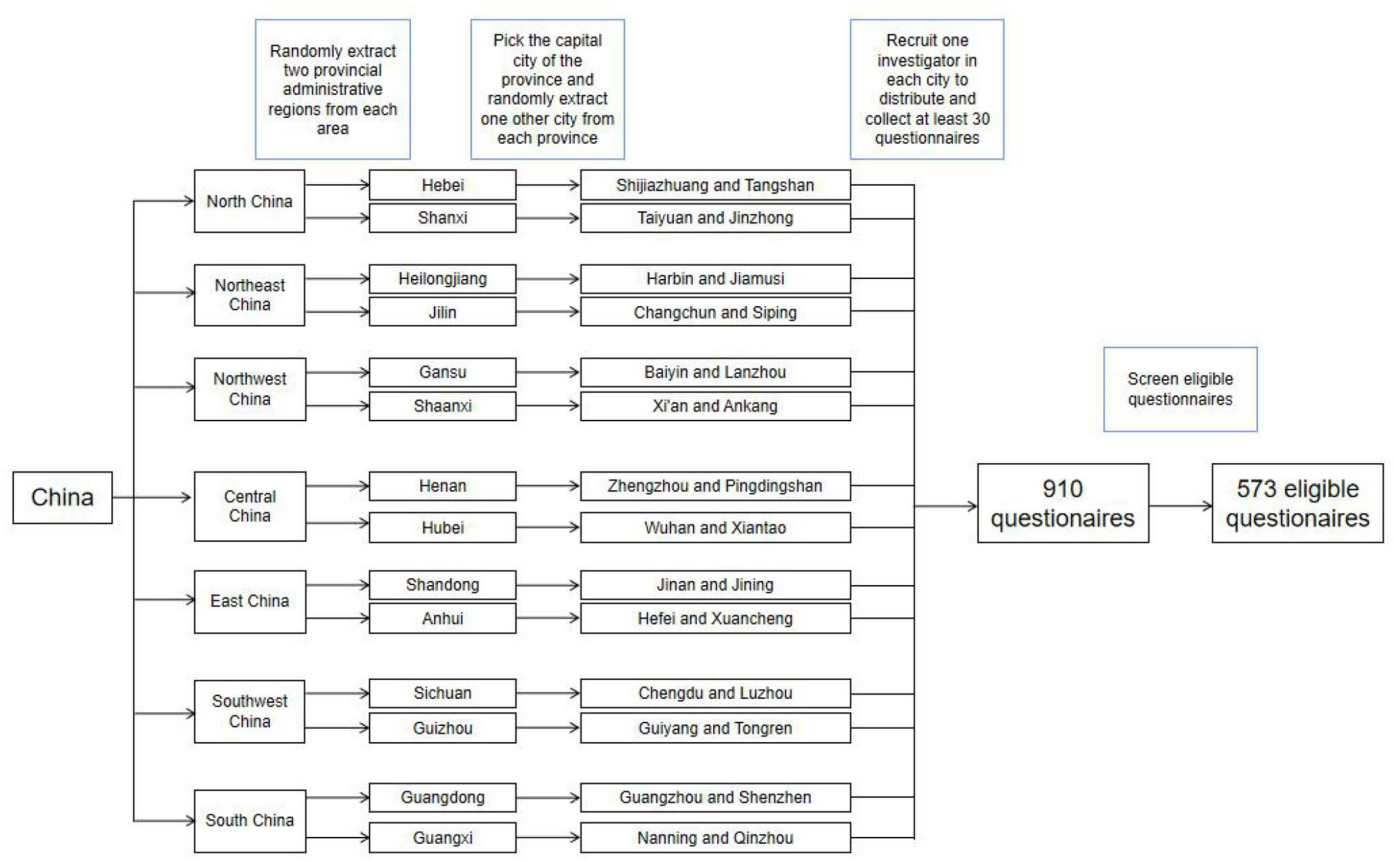
Sampling and screening process of qualified questionnaires.

Multi-stage stratified sampling was used (see Figure 2 for details). There were 7 administrative regions in China (North China, Northeast China, East China, Central China, South China, Southwest China, and Northwest China). Of the 7 administrative regions, two provinces were firstly randomly selected from each (excluding Hong Kong, Macao, and Taiwan). Then the provincial capital cities of each provincial administrative region were selected, and then another city was randomly selected in each provincial administrative region (if it was a municipality directly under the central government, Beijing, Shanghai, Chongqing and Tianjin, this step would be skipped). Twenty-eight cities were selected (1 provincial capital city and 1 prefecture-level city in each province). The selected cities were as follows: Jinan and Jining in Shandong Province, Hefei, and Xuancheng in Anhui Province, Baiyin and Lanzhou in Gansu Province, Guangzhou and Shenzhen in Guangdong Province, Nanning and Qinzhou in Guangxi Province, Guiyang and Tongren in Guizhou Province, Shijiazhuang and Tangshan in Hebei Province, Zhengzhou and Pingdingshan in Henan Province, Harbin and Jiamusi in Heilongjiang Province, Wuhan and Xiantao in Hubei Province, Changchun and Siping in Jilin Province, Taiyuan and Jinzhong in Shanxi Province, Xi'an and Ankang in Shaanxi Province, Chengdu and Luzhou in Sichuan Province. One volunteered investigator was recruited in each city, each of whom was required to distribute and collect at least 30 questionnaires by sending the questionnaire link online or forwarding the questionnaire link face-to-face. All questionnaires were produced and distributed through the Wenjuanxing platform (https://www.wjx.cn/). Before starting the investigation, investigators were trained in standardization, and then they investigated by forwarding questionnaire links to those who met the inclusion criteria one by one. The period of questionnaire distribution was from August 15, 2020, to September 5, 2020.

### Ethics Review

This research has passed the ethical review of Shaanxi Health Culture Research Center, the ethical review document is JKWH-2020-17.

### Quality Control

The questionnaire for expert consultation was designed. According to the scores of the experts in the expert consultation, the content validity indexes (I-CVI and S-CVI/Ave) of the questionnaire were calculated. The expert group consists of eight pharmacists with bachelor's degrees or above and working in a secondary or tertiary hospital. Also, a pilot study was conducted online. We recruited participants online to participate in the pilot study, and 37 participants who met the inclusion criteria participated in it. Items of the questionnaire were revised according to the pilot study data and expert opinions. The reliability and validity of the questionnaire were tested before the questionnaire was issued, and the final questionnaire was settled after excluding irrelevant items. Before the formal investigation, the investigators were trained uniformly to ensure the standardization of the questionnaire distribution process. Supervisors filled out the questionnaire anonymously to reduce their concerns about privacy leakageand ensure the data's authenticity as much as possible. After the questionnaires were retrieved, invalid questionnaires were first excluded through verification, including questionnaires that took <1 min to answer and questionnaires with inconsistent content.

### Statistical Method

SPSS 21.0 was used to test the reliability and validity of the questionnaire, and the Cronbach'α and split-half reliability values were used to evaluate the reliability of the questionnaire. I-CVI and S-CVI/Ave (Average value of I-CVI) were used to test the content validity. The KMO value and Bartlett's spherical test were used to determine whether questionnaire items were factor-analyzed. After the questionnaires were collected, the valid data were imported into SPSS 21.0 for statistical analysis. Mean ± standard derivation was used to describe the score of medication adherence. IBM AMOS 24.0 was used to establish the structural equation model. The score of the medication adherence tool is used as the dependent variable in the structural equation model. The relationship between the other 6 dimensions (perceived susceptibility, perceived severity, perceived benefits, perceived barriers, self-efficacy, objective constraints) and their influence on the dependent variable is determined by structural equation modeling. In addition to establishing structural equation models for all parents, four structural equation models were established for male, female, urban, and rural parents for subgroup analysis to study the differences between parents with different characteristics. The common evaluation indicators for the rationality of the structural equation model are Chi-square/DF (Chi-squared over degrees of freedom), GFI (Goodness of Fit Index), AGFI (Adjusted Goodness of Fit Index), CFI (Comparative Fit Indexes), RMSEA (Root Mean Square Error of Approximation), with α = 0.05 as the statistical test standard for significance.

## Results

This study finally retrieved 910 questionnaires, of which 573 were valid, with an effective rate of 62.97%. The questionnaire distribution process is shown in Figure 2. Due to the impact of the COVID-19 epidemic, this study was conducted by online survey, which may be the reason for the relatively low efficiency of the questionnaires.

### Reliability and Validity Test of the Questionnaire

The questionnaire's Cronbach coefficient and split-half reliability coefficient were, respectively, 0.821 > 0.6 and 0.651 > 0.6, indicating that the questionnaire had good reliability. The I-CVI of the items of health belief model dimensions are all higher than 0.78, and the S-CVI/Ave (Average value of I-CVI) is 0.95 > 0.9, which proves it has good content validity. The questionnaire KMO value was 0.875 > 0.8, and the Bartlett sphere test *p* < 0.001, indicating that it is suitable for factor analysis. After exploratory factor analysis, the health belief model questionnaire can be divided into 6 dimensions, the dimensional division is basically consistent with the theoretical framework, and the cumulative variance explanation rate is 58.19% (>50%), indicating good construct validity of the questionnaire.

### Demographic Characteristics of the Parents and the Medication Adherence of the Children Cared for by the Parents

The score of the medication adherence tool was 12.56 ± 1.07 (mean ± SD). Parents with 13–14 points (good medication adherence of children) accounted for 57.24%, 12 points (general medication adherence of children) accounted for 27.40%, 11 points and below (poor medication adherence of children) accounted for 15.36%. The demographic sociological characteristics of the sample population and the score of children' medication adherence of respondents are shown in Table 2.

**Table 2:** Demographic characteristics and score of children's medication adherence of respondents.

**Item**	**Option**	**Number**	**Score of medication**
		**(percentage)**	**adherence(mean ±SD)**
Gender	Male	178 (31.06 %)	12.44 ± 1.10
	Female	395 (68.94%)	12.62 ± 1.06
Age group	21–30	164 (28.62%)	12.66 ± 1.04
	31–40	318 (55.50%)	12.49 ± 1.10
	Over 40	91 (15.88%)	12.60 ± 1.04
Per capita GDP level of the province where the respondent is located	Lower than the average	223 (38.92%)	12.71 ± 0.92
	Higher than the average	350 (61.08%)	12.47 ± 1.15
Education level	Junior middle school and below	139 (24.26%)	12.48 ± 1.26
	Technical secondary school	45 (7.85%)	12.62 ± 1.03
	Senior middle school	52 (9.08%)	12.60 ± 1.02
	Junior college	78 (13.61%)	12.59 ± 1.09
	Undergraduate	208 (36.30%)	12.61 ± 0.97
	Postgraduate	51 (8.90%)	12.45 ± 1.03
Number of children in the family	1	289 (50.44%)	12.64 ± 1.01
	More than 1	284 (42.58%)	12.48 ± 1.13
Domicile	urban	388 (67.71%)	12.51 ± 1.06
	rural	185 (32.29%)	12.66 ± 1.10
Monthly income per capita in the household (Unit: USD)	Above 611	195	12.56 ± 1.17
	611–917	165	12.56 ± 1.03
	917–1,222	84	12.49 ± 1.04
	More than 1,222	129	12.60 ± 1.01

*SD, Standard derivation*.

### Structural Equation Model of the Factors Affecting Medication Adherence in Children Cared for by the Parents

The independent variable of this study was the score of objective constraints. The dependent variables of this study were the score of perceived susceptibility, perceived severity, perceived benefits, perceived barriers, self-efficacy, and medication adherence. The outcome variable of this study was the score of medication adherence. The KMO value (0.875) and the Bartlett sphere test result (*p* < 0.001) indicate that the data is suitable for the construction of the structural equation model. According to the theory of the health belief model and the index of model fit, the six dimensions (perceived severity, perceived benefits, perceived barriers, perceived susceptibility, self-efficacy, and objective constraints) determined during the questionnaire design are all included as independent variables into the structural equation model. The model dependent variable is the score of the children's medication adherence assessment tool. The structural equation model of all parents has been revised several times to fit the best model of factors affecting medication adherence. The fitting indices are as follows: CMIN/DF = 2.334 < 3, GFI = 0.992 > 0.9, AGFI = 0.968 > 0.9, CFI = 0.986 > 0.9, RMSEA = 0.048 < 0.08, NFI = 0.977 > 0.9, TLI = 0.959 > 0.9, IFI = 0.986 > 0.9, indicating that the model fits the data, and the model is scientific and effective. The fitting indices of the model for male parents are as follows: CMIN/DF = 1.647 < 3, GFI = 0.982 > 0.9, AGFI = 0.927 > 0.9, CFI = 0.983 > 0.9, RMSEA = 0.060 < 0.08, NFI = 0.960 > 0.9, TLI = 0.950 > 0.9, IFI = 0.984 > 0.9. The fitting indices of the model for female parents are as follows: CMIN/DF = 1.723 < 3, GFI = 0.991 > 0.9, AGFI = 0.965 > 0.9, CFI = 0.987 > 0.9, RMSEA = 0.043 < 0.08, NFI = 0.971 > 0.9, TLI = 0.961 > 0.9, IFI = 0.988 > 0.9. The fitting indices of the model for urban parents are as follows: CMIN/DF = 1.339 < 3, GFI = 0.993 > 0.9, AGFI = 0.973 > 0.9, CFI = 0.994 > 0.9, RMSEA = 0.030 < 0.08, NFI = 0.978 > 0.9, TLI = 0.983 > 0.9, IFI = 0.994 > 0.9. The fitting indices of the model for rural parents are as follows: CMIN/DF = 2.403 < 3, GFI = 0.975 > 0.9, AGFI = 0.900 = 0.9, CFI = 0.963 > 0.9, RMSEA = 0.087 > 0.08, NFI = 0.941 > 0.9, TLI = 0.889 < 0.9, IFI = 0.965 > 0.9. The models for male, female and urban parents fit the data and are all scientific and effective. Several model fit metrics in the model for rural parents are slightly out of specification, but the overall quality of the model is good and the model can be used for further analysis. Scores of dimensions in structural equation model are shown in Table 3.

**Table 3:** Scores of dimensions in structural equation model.

**Dimension**	**No. of**	**Total**	**Mean**	**Standard**
	**items**	**score**		**deviation**
Perceived susceptibility	4	20	9.55	2.56
Perceived severity	5	25	19.30	3.34
Perceived benefits	3	15	11.88	1.79
Perceived barriers	7	35	18.54	5.10
self-efficacy	3	15	11.62	1.79
Objective constraints	4	20	12.68	2.54
Medication adherence of children	7	14	12.56	1.07

**Figure 3:**
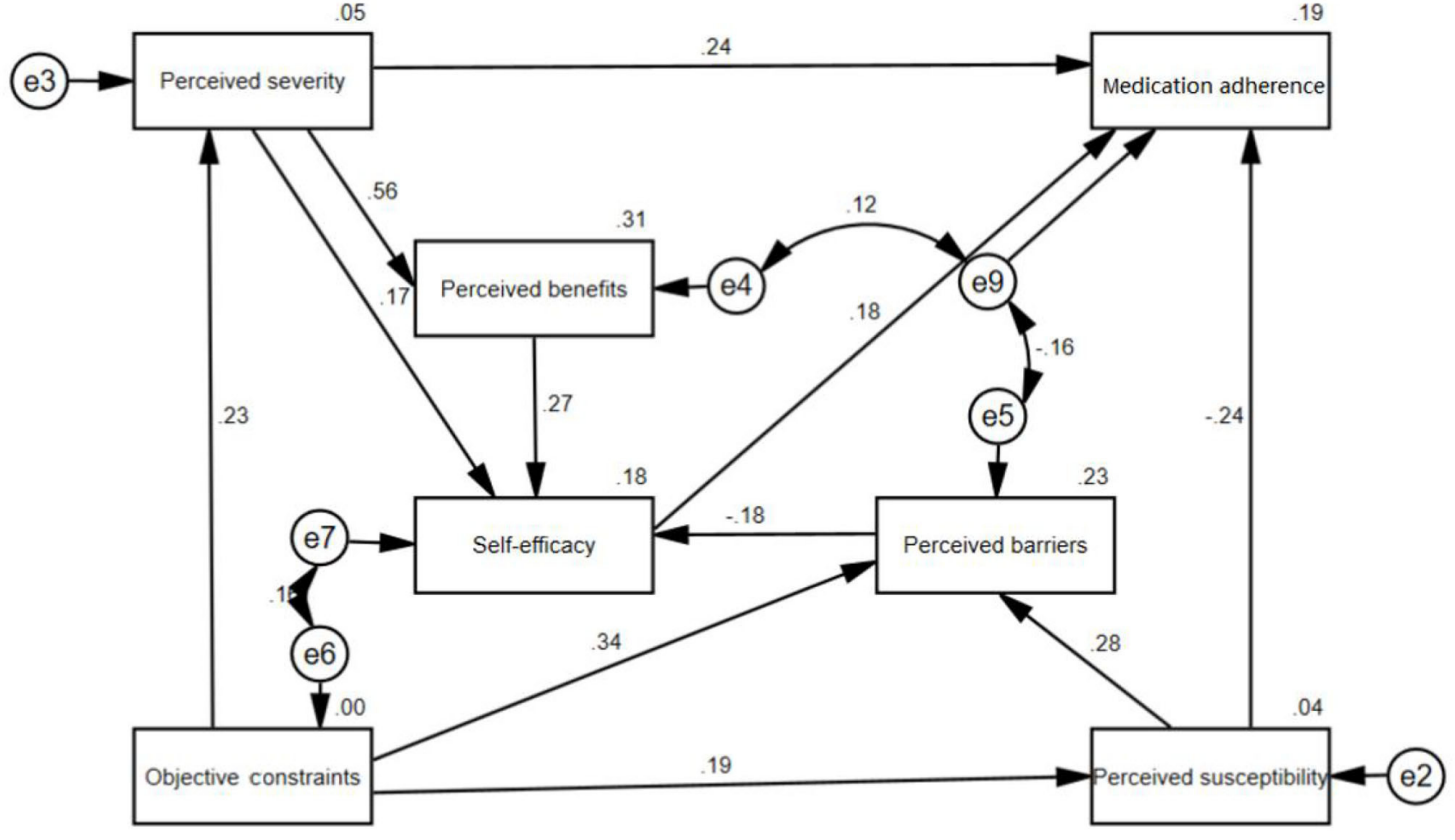
Structural equation model of parents' health belief on their children's medication adherence. The value on the one-way arrow is the coefficient in the structural equation model, indicating the magnitude of the former's influence on the latter. If the value on the one-way arrow is positive, it means that the former has a positive influence on the latter. If the value on the one-way arrow is negative, it means that the former has a negative influence on the latter. e2, e3, e4, e5, e6, e7, e8, and e9 are residual items, representing confounding factors independent of the established structural equation model.

In Figure 3, the structural equation model shows that self-efficacy (λ = 0.177, P < 0.001) and perceived severity (λ = 0.243, P < 0.001) have significant positive direct impacts on medication adherence. Perceived susceptibility (λ = −0.244, *P* < 0.001) has a significant negative direct impact on medication adherence. The indirect effects on medication adherence are as follows: objective constraints (λ = 0.009, *P* < 0.001), perceived barriers (λ = −0.033, *P* < 0.001), perceived benefits (λ = 0.049, *P* < 0.001).

**Figure 4:**
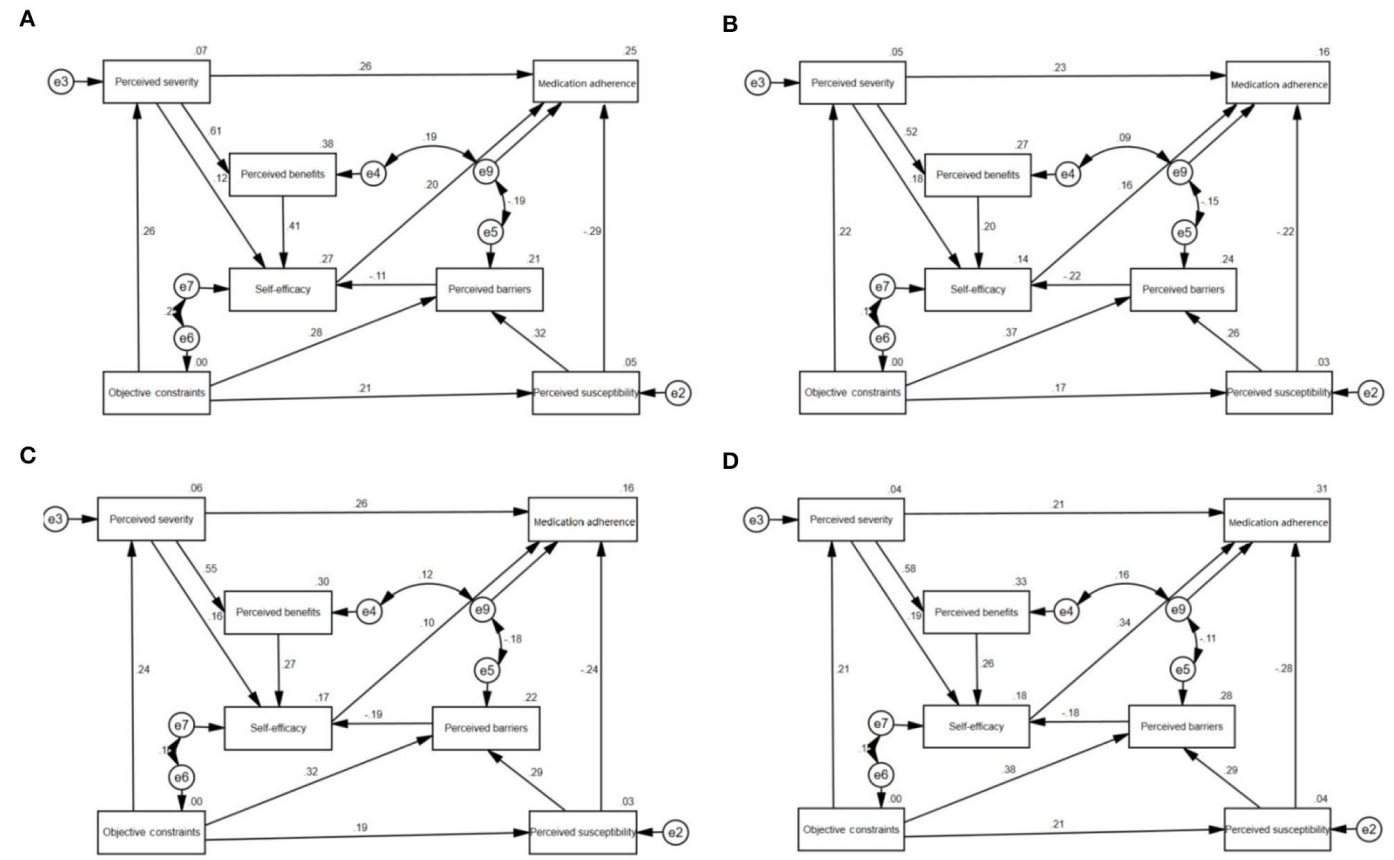
Structural equation model of female, male, urban and rural parents' health belief on their children's medication adherence **(A)** female **(B)** male **(C)** urban **(D)** rural.

In the subgroup analysis, the model established by each subgroup is basically consistent with the model established by the overall sample, but there are differences in some coefficients (Figure 4, Tables 4, 5). The results of subgroup analysis of dimension coefficients that have a direct impact on children's medication adherence are shown in Tables 4, 5. The absolute values of the coefficients of each dimension directly related to the medication adherence of female parents are greater than those of male parents. The absolute values of the coefficients of perceived susceptibility and self-efficacy of rural parents are greater than those of urban parents, while the absolute value of the coefficient of perceived severity of rural parents is greater than that of urban parents.

**Table 4:** Coefficients of each dimension directly related to children's medication adherence between parents of different genders.

	**Perceived susceptibility**	**Perceived severity**	**Self-efficacy**
Male	−0.24	0.24	0.18
Female	−0.29	0.26	0.20

**Table 5:** Coefficients of each dimension directly related to children's medication adherence between parents of different places of residence.

	**Perceived susceptibility**	**Perceived severity**	**Self-efficacy**
urban	−0.24	0.26	0.10
Village	−0.28	0.21	0.34

## Discussion

### Analysis of Medication Adherence of Children Under the Care of Parents

Several studies (28–31) have shown that after a child becomes ill, the main caregivers are mostly women, which is why women accounted for a relatively large proportion (68.94%) in this study. According to the results of the medication adherence tool, the proportion of the supervisors' children with good medication adherence was 57.24% (medication adherence tool scores 13 or 14), the general proportion was 27.40% (medication adherence tool scores 12), and the poor proportion was 15.36% (medication adherence tool scores 11 or less). However, in this online study, we did not ask each participant the exact number of children, so we were unable to obtain the proportions of children with good, moderate, or poor medication adherence. Vasylyeva et al. (18) investigated the medication adherence of children with chronic kidney disease who had taken at least three medications for more than 3 months, and found that 41.1% of the children did not adhere to the doctor's advice. Miner et al. (32) conducted a cross-sectional study on the medication adherence of 100 children with epilepsy between 2 and 14 years old and found that only 28% of the children had good medication adherence. Nazziwa et al. (33) used two methods (self-report method and blood drug concentration monitoring method) to evaluate the medication adherence of 122 children with epilepsy, and the results showed that the medication adherence from the self-report method was 79.5%, which was higher than the results of this study, which may be related to the different questionnaires used and cultural differences. However, the medication adherence obtained by monitoring the blood concentration was 22.1%, which was lower than the results of this study. Due to the large differences in the time, place, method and investigator's disease status of each study, the results obtained by different studies are also quite different.

### Analysis of the Structural Equation Model of All Parents

Various methods had been used to measure the reliability and validity of the questionnaire, including Cronbach's coefficient, split-half reliability, content validity, exploratory factor analysis, and confirmatory factor analysis. The conduct of the pilot study and expert consultation also ensures the reliability and validity of the questionnaire and the reliability of the research results. The structural equation model shows that parents' perceived susceptibility (λ = −0.244, *P* < 0.001), perceived severity (λ = 0.243, *P* < 0.001), and self-efficacy (λ = 0.177, *P* < 0.001) have significant direct effects on children's medication adherence. The direct effect of perceived susceptibility is negative. Perceived susceptibility refers to subjective assessment of the risk of developing a health problem. The children of parents with higher scores on the perceived susceptibility dimension have a higher risk of non-adherence with medication. In past studies, perceived susceptibility was often positively correlated with subjects' medication adherence (34, 35). Many studies explored medication adherence in patients with chronic diseases such as hypertension and tuberculosis. In these studies, perceived susceptibility is often defined as the susceptibility to the onset or progression of a disease. However, our study focused on parents whose children had different diseases. In our study, perceived susceptibility is defined as the susceptibility of children's non-adherence to medication. The above two reasons are the main difference between our research and other research results.

Perceived severity has a direct positive effect which is the parents' subjective perception of potential harm. When the parents realize that non-adherence to medication will damage children's health, they will be more likely to follow the doctor's advice. Many other studies have also shown that perceived severity is positively correlated with patient adherence to treatment (34–36).

Self-efficacy refers to an individual's perception of his or her competence to successfully perform a behavior (37). Multiple studies have shown that patients with chronic diseases (such as hypertension, type 2 diabetes, and tuberculosis) with higher levels of self-efficacy tend to have higher treatment adherence (38–42). Parents with higher scores on the self-efficacy dimension have the higher subjective initiative in improving children's medication adherence, so their children's medication adherence scores are relatively higher, which suggests that improving parents' self-efficacy may be an effective way to improve children's medication adherence.

In addition, there is a wide range of mediating effects in the model, perceived benefits (λ = 0.049, *P* < 0.001), perceived barriers (λ = −0.033, *P* < 0.001), and objective constraints (λ = 0.009, *P* < 0.001) all have indirect effects on medication adherence. Perceived severity, perceived benefits, and perceived barriers can all affect self-efficacy and thereby affect the medication adherence of the children. Objective constraints and perceived benefits have a positive indirect effect on medication adherence, while perceived barriers have a negative indirect effect on medication adherence. It can be seen from the coefficients that among the three indirect influencing factors of medication adherence in the model, perceived barriers and perceived benefits have greater impacts on medication adherence. Perceived benefits refer to parents' perceptions of the benefits of good medication adherence in their children. The more parents are aware of this kind of benefits, the more they will pay attention to the improvement of their children's medication adherence. Instead, perceived barriers refer to barriers that parents have in improving their children's medication adherence. The greater the difficulties parents encounter, the worse their children's medication adherence would be. In multiple studies on patient treatment adherence, both perceived benefits and perceived barriers have opposite effects on patients' treatment adherence (43–45), which is consistent with the findings of this study.

### Analysis of the Structural Equation Model of the Four Subgroups

In the subgroup analysis, the absolute values of females' perceived susceptibility, perceived severity, and self-efficacy for their children's medication adherence path coefficients were higher than those of males, which may be related to females' family responsibilities and psychological characteristics. In Chinese families, mothers are usually the main caregivers of their children. Compared with fathers, mothers are more concerned about their children's physical and mental health, and they often put more effort into the care of children (46, 47). Therefore, females' health beliefs and their children's medication adherence are more closely. In terms of urban-rural differences, the absolute value of the path coefficient of the children's medication adherence with perceived susceptibility and self-efficacy of the rural parents was higher than that of the urban parents, while the absolute value of the perceived severity of their children's medication adherence path coefficient was lower than that of the urban parents. The reasons are as follows. First, the health literacy and medication literacy of parents in rural areas may be lower than those in urban areas, which makes it difficult for them to recognize the adverse consequences caused by their children's poor medication adherence (48, 49). Therefore, the absolute value of the perceived severity of their children's medication adherence path coefficient is lower than that of the urban parents. Secondly, the parents in rural areas are less busy than those in urban areas, so they have more time to take care of their children. Therefore, the absolute values of the perceived susceptibility and self-efficacy of the rural parents to their children's medication adherence path coefficient are higher than those of the urban parents.

### Suggestions

The results of structural equation modeling suggested that the perceived severity of parents' non-adherence with drug use by their children had a positive impact on their children's adherence to drug use (50). Medical staff should explain medication adherence knowledge to the parents of children, inform the children of the possible consequences of non-adherence with medication, and improve the understanding of children and their families on the hazards of non-adherence with medication, to improve the subjective perception of parents on the severity of children's non-adherence with medication.

Self-efficacy is the behavioral belief of parents about whether they can achieve the goal of children's good medication adherence. The higher the self-efficacy score is, the more parents can help children maintain good medication behavior (51). Therefore, in order to improve children's medication adherence, we should focus on improving parents' self-efficacy of rational medication for children. Health care personnel should ensure good communication with parents, explain in easy-to-understand language items needing attention in children's medication process, such as medication times, medication time, and adverse reactions, and provide medication guidance. In addition, medical institutions can hold regular parents' communication meetings to encourage parents to participate, encourage each other actively, and share children's medication experiences to improve parents' self-efficacy in rational medication for children.

In addition, attention should be paid to the mental health of the parents of children, and more social and psychological support should be provided for the parents to help them solve the problems in the process of children's medication. Multiple studies have shown that the better the psychological state of the parents, the stronger their self-efficacy, and this study shows that high parental self-efficacy can help improve children's medication adherence (52–54). Only by strengthening the self-psychological construction of the parents of children and giving them more effective social and psychological support can the parents play a greater role in family education, help children to establish good drug habits, and play an important role in promoting the mental health of children (55, 56).

### Advantages and Limitations

The advantages of the study are as follows. Firstly, this is a China-wide cross-sectional investigation, and the selected sample is representative to a certain extent, which makes the research results more credible. Secondly, from a research perspective, this study innovatively proposes that parents' health beliefs may have an impact on their children's medication compliance and conducts an in-depth study of this. The limitations of the study are as follows. Firstly, as the research data is in the form of self-reporting, it is difficult to eliminate some recall bias. Secondly, this was cross-sectional research, so the conclusions are only the dimensional factors of the current health belief model and the parents' children medication adherence relationship. This study only explored the relationship between parents' health beliefs and their children's medication adherence and could not clarify the causal link between parents' health beliefs and their children's medication adherence, which needs further investigation. Due to the impact of the COVID-19, offline questionnaire investigations cannot be conducted, so the convenience of sampling by investigators cannot be completely randomized. Inevitable selection bias may exist, which may affect the results. Finally, the efficiency of the questionnaire in this study is not high, which may be related to factors such as the survey method and questionnaire design, and we will improve it in future research.

## Conclusions

Parents have an impact on their children's medication adherence. From the structural equation model, the parents' perceived severity and self-efficacy may have positive direct effects, while parents' perceived susceptibility may have a negative direct effect on their children's medication adherence. Perceived benefits and objective constraints may have a positive indirect effect on their children's medication adherence, while perceived barriers may have a negative indirect effect on their children's medication adherence. Women's health belief appears to have a greater impact on their children's medication adherence than men's. It may be an effective strategy to increase their children's medication adherence by improving parents' health beliefs.

## Prospect

In the practice of pediatric pharmacy, it is necessary to strengthen the medication education for parents so that they can realize the importance of their children's medication adherence and the serious consequences of non-adherence, then their self-efficacy would be improved to improve children's medication adherence.

## Data Availability Statement

The original contributions presented in the study are included in the article/Supplementary Material, further inquiries can be directed to the corresponding authors.

## Ethics Statement

The studies involving human participants were reviewed and approved by the ethical review of Shaanxi Health Culture Research Center, the ethical review document is JKWH-2020-17. The patients/participants provided their written informed consent to participate in this study.

## Author Contributions

Y-bW, PG, and S-tL: conceptualization. PG: data curation. PG, S-xX, and Y-jL: formal analysis. PG, S-tL, and J-zZ: investigation. Y-bW, PG, and S-tL: methodology. Y-bW and PG: project administration. Y-bW and YB: resources. Y-bW: supervision. PG, S-xX, and R-cF: visualization. PG and J-zZ: writing—original draft. Y-bW, YB, PG, S-xX, X-yK, and J-zZ: writing—review and editing. All authors have read and agreed to the published version of the manuscript.

## Funding

This study was supported by the Science and Technology Development Center of the Chinese Pharmaceutical Society Fund (Grand: CMEI2021KPYJ00607) and the research funds of Ying Bian from the University of Macau (Grand: MYRG2019-00044-ICMS).

## Conflict of Interest

The authors declare that the research was conducted in the absence of any commercial or financial relationships that could be construed as a potential conflict of interest.

## Publisher's Note

All claims expressed in this article are solely those of the authors and do not necessarily represent those of their affiliated organizations, or those of the publisher, the editors and the reviewers. Any product that may be evaluated in this article, or claim that may be made by its manufacturer, is not guaranteed or endorsed by the publisher.
