# Supplementary material for: The Influence of Parents on Medication Adherence of Their Children in China: A Cross-Sectional Online Investigation Based on Health Belief Model
Source: Front Public Health. 2022 Apr 14;10:845032. doi: 10.3389/fpubh.2022.845032 (PMC9046660; doi:10.3389/fpubh.2022.845032)
Supplement: Supplementary file 1 [file Data_Sheet_1.ZIP › Supplementary_Material/Figures and Tables.docx]

**Figures and Tables**

Table 1. Demographic characteristics of respondents

| **Item** | **Option** | **Number of people who chose this option** | **Percentage (%)** |
| --- | --- | --- | --- |
| Gender | Male | 178 | 31.06 |
|  | Female | 395 | 68.94 |
| Age group | 21-30 | 164 | 28.62 |
|  | 31-40 | 318 | 55.50 |
|  | Over 40 | 91 | 15.88 |
| Per capita GDP level of the province where the respondent is located | lower than the average | 223 | 38.92 |
|  | Lower than the average | 350 | 61.08 |
| Education level | Junior middle school and below | 139 | 24.26 |
|  | Technical secondary school | 45 | 7.85 |
|  | Senior middle school | 52 | 9.08 |
|  | Junior college | 78 | 13.61 |
|  | Undergraduate | 208 | 36.30 |
|  | Postgraduate | 51 | 8.90 |
| Number of children in the family | 1 | 289 | 50.44 |
|  | More than 1 | 284 | 42.58 |
| Domicile | Town | 388 | 67.71 |
|  | Village | 185 | 32.29 |
| Monthly income per capita in the household (Unit: USD) | Above 611 | 195 | 34.03 |
|  | 611-917 | 165 | 28.80 |
|  | 917-1222 | 84 | 14.66 |
|  | More than 1222 | 129 | 22.51 |


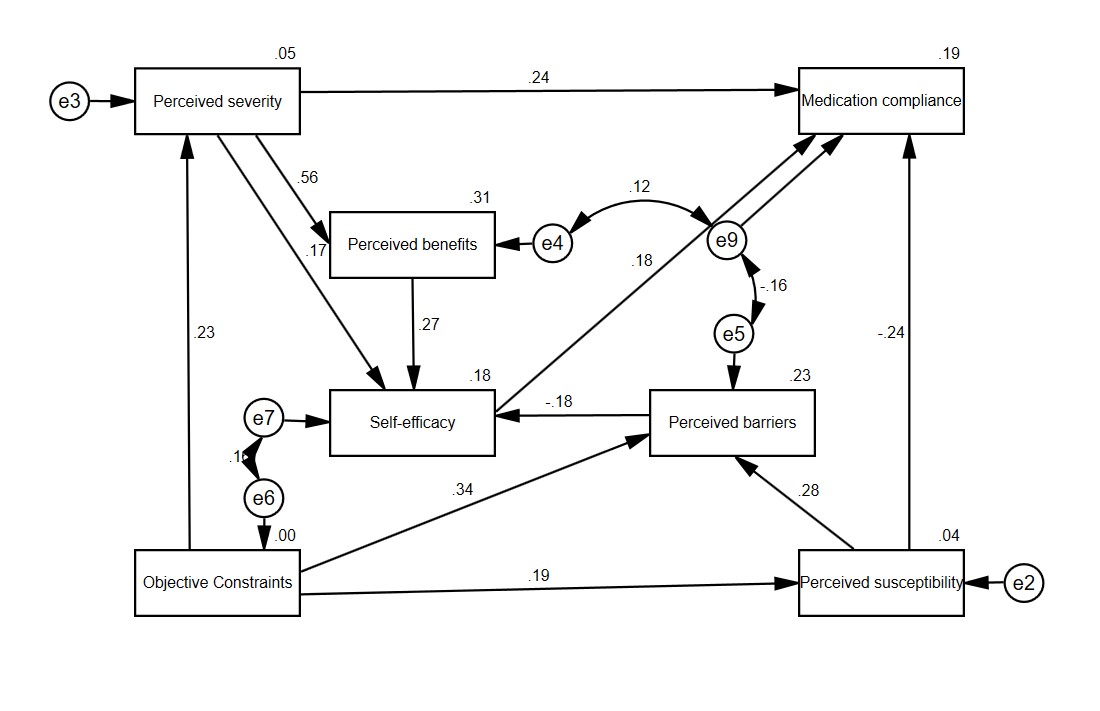


Figure 1. Structural equation model of supervisor's influence on their children's medication adherence
