# Supplementary material for: The Influence of Parents on Medication Adherence of Their Children in China: A Cross-Sectional Online Investigation Based on Health Belief Model
Source: Front Public Health. 2022 Apr 14;10:845032. doi: 10.3389/fpubh.2022.845032 (PMC9046660; doi:10.3389/fpubh.2022.845032)
Supplement: Supplementary file 1 [file Data_Sheet_1.ZIP › Supplementary_Material/Medical Ethics Committee Approval Form.docx]

Medical Ethics Committee Approval Form

**serial number
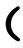
*No*.
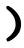
** **:JKWH-2020-17**

| 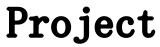 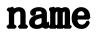 | The influence of supervisor on medication adherence of their children in China：an online investigation based on health belief model | | | | | |
| --- | --- | --- | --- | --- | --- | --- |
| **Category** | □Human specimen collection  ☑ Subject data collection   - New technology clinical application - New drug clinical trials - other： | | | | | |
| **Research period** | July 1, 2020 | | To date | June 30, 2021 | | |
| **Project manager** | Wu Yibo | **Gender** | Male | | **job title** | researcher |
| **research direction** | health education | **contact details** | 18810169630 | | **E-mail** | bjmuwuyibo@outloo k.com |
| **project source** | □National Natural Science Foundation ofChina  □Natural Science Foundation of Shaanxi Province  □Shaanxi Provincial Department of Science and Technology  □Shaanxi Provincial Department ofEducation  □Shaanxi Provincial Health Commission  □On campus  ☑ Other： Self-developed project | | | | | |
| **research content**  **Summary** | (The summary of research content mainly includes: project background and purpose, subject selection, control settings, intervention measures, observation indicators, follow-up status, sample size, statistical analysis, risk/benefit analysis, subject protection measures, etc.)  Project background and purpose: Use the adapted Children’s Medication Compliance Scale (MG) to conduct a questionnaire survey on the parents of children aged 0-12 to reveal the current status of children’s medication compliance; use the Health Belief Model (HBM) as the theoretical framework to develop factors affecting children’s medication compliance The questionnaire provides a reliable tool for parents to objectively evaluate the influencing factors of children's medication compliance; construct a structural equation model to explore the impact of parent factors on children's medication compliance, and develop effective interventions for targeted health education activities for parents Measures provide a basis.  Participants' inclusion criteria: 1. Parents with children ≤12 years old 2. Informed consent and voluntary participation in this study 3. Mainly responsible for the medication of children in the family  Investigation method: Taking the parents of children aged 0-14 as the research object, according to the principle of multi-stage stratified cluster sampling, and according to the characteristics of China's geographical distribution, sampling from each of the 7 administrative regions of North China, Northeast China, East China, Central China, South China, Southwest China, and Northwest China2 A total of 14 provinces (autonomous regions, municipalities directly under the Central Government), and then 2 cities (1 provincial capital city, 1 prefecture-level city) from the extracted provinces, skip this step if it is a municipality directly under the Central  Government. Recruit one investigator in each city, and determine the number of | | | | | |

|  | questionnaires to be issued for each investigator based on the minimum sample size calculated from the pre-survey data.  Control setting: This study adopts the method of cross-sectional study, and there is no control setting.  Intervention measures: There are no intervention measures in this study. Observation indicators: medication compliance.  Follow-up: This study does not involve follow-up. Sample size: Approximately 1,000 questionnaires were collected.Observation indicator: healthy behavior.  Statistical analysis: This study will use SPSS and AMOS software to conduct statistical analysis of effective questionnaires.  Risk/benefit analysis: The issuance of this questionnaire mainly relies on the participants' interest in participating in voluntary filling. This study can understand the current status of children's medication compliance, so as to provide a basis for relevant departments to carry out targeted health education and improve patient health management. The research team will promptly disclose the results of the paper to guide the promotion of healthy behaviors.  Participant protection measures: This survey adopts an anonymous method, and the question design does not involve personal privacy. |
| --- | --- |
| **Review method** | □Quick review Meeting review  □Emergency meeting review |


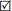


| **Basis for review** | ☑ Test plan  ☑ Informed consent  ☑ Researcher's CV  □ Safety measures and emergency plans  other information See attached questionnaire  (Other materials include: safety materials oftest supplies, qualification certificates ofmanufacturers, and qualification certificates of test supplies providers) |
| --- | --- |
| **Project manager committed to** | 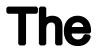 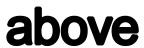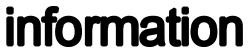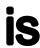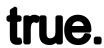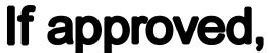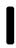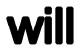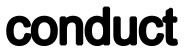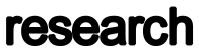 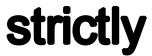 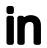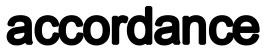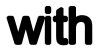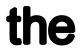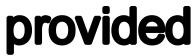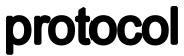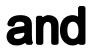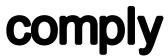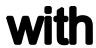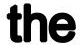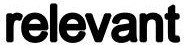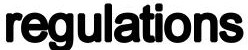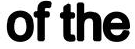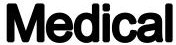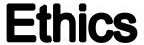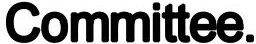 : |
| **Review result** | Agree to the application.  ☑agree □disagree  □Agree after making necessary changes |
| **Review conclusion** | According to the experimental design of the study, the health, rights and privacy of the subjects are fully protected after review by the ethics  committee, and the potential risks and harm to the subjects can be controlled to  a minimum. |
| **Signature of Scientific Research Office:**  Research Office of Shaanxi Institute of International Business  Date: June 2020 | |

Note: 1. This approval form should be printed on both sides, in duplicate, and submitted to the ethics committee.


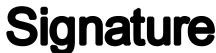

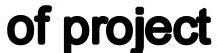

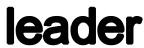


2. At the same time, submit two copies of the detailed information of the review basis for review by the reviewer.
